# Neurons Derived from Human Induced Pluripotent Stem Cells Integrate into Rat Brain Circuits and Maintain Both Excitatory and Inhibitory Synaptic Activities

**DOI:** 10.1523/ENEURO.0148-19.2019

**Published:** 2019-08-22

**Authors:** Xiling Yin, Jin-Chong Xu, Gun-sik Cho, Chulan Kwon, Ted M. Dawson, Valina L. Dawson

**Affiliations:** 1 Neuroregeneration and Stem Cell Programs, Institute for Cell Engineering, Johns Hopkins University School of Medicine, Baltimore, Maryland 21205; 2 Department of Neurology, Johns Hopkins University School of Medicine, Baltimore, Maryland 2120; 3 Division of Cardiology, Department of Medicine, Johns Hopkins University School of Medicine, Baltimore, Maryland 21205; 4 Department of Physiology, Johns Hopkins University School of Medicine, Baltimore, Maryland 21205; 5 Soloman H. Snyder Department of Neuroscience, Johns Hopkins University School of Medicine, Baltimore, Maryland 21205; 6 Department of Pharmacology and Molecular Sciences, Johns Hopkins University School of Medicine, Baltimore, Maryland 21205; 7 Adrienne Helis Malvin Medical Research Foundation, New Orleans, Louisiana 70130; 8 Diana Helis Henry Medical Research Foundation, New Orleans, Louisiana 70130

**Keywords:** balanced excitatory and inhibitory network, hiPSC-derived neurons

## Abstract

The human cerebral cortex is a complex structure with tightly interconnected excitatory and inhibitory neuronal networks. In order to study human cortical function, we recently developed a method to generate cortical neurons from human induced pluripotent stem cells (hiPSCs) that form both excitatory and inhibitory neuronal networks resembling the composition of the human cortex. These cultures and organoids recapitulate neuronal populations representative of the six cortical layers and a balanced excitatory and inhibitory network that is functional and homeostatically stable. To determine whether hiPSC-derived neurons can integrate and retain physiologic activities *in vivo*, we labeled hiPSCs with red fluorescent protein (RFP) and introduced hiPSC-derived neural progenitors to rat brains. Efficient neural induction, followed by differentiation resulted in a RFP^+^ neural population with traits of forebrain identity and a balanced synaptic activity composed of both excitatory neurons and inhibitory interneurons. Ten weeks after transplantation, grafted cells structurally integrated into the rat forebrain. Remarkably, these hiPSC-derived neurons were able to fire, exhibiting both excitatory and inhibitory postsynaptic currents, which culminates in the establishment of neuronal connectivity with the host circuitry. This study demonstrates that neural progenitors derived from hiPSCs can differentiate into functional cortical neurons and can participate in neural network activity through functional synaptic integration *in vivo*, thereby contributing to information processing.

## Visual Abstract

**Figure:**
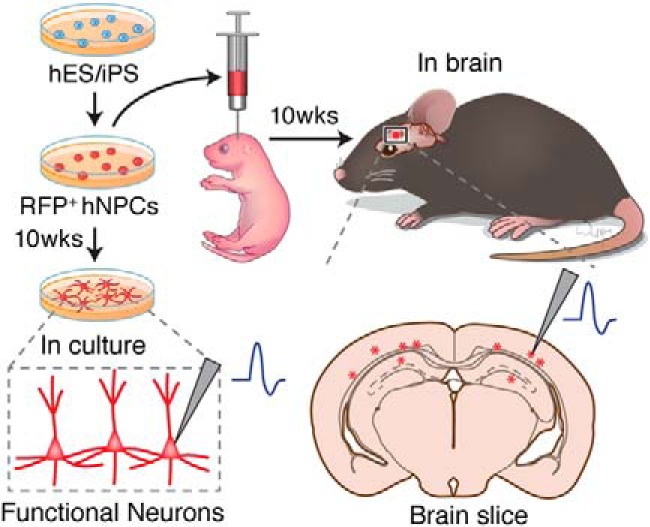

## Significance Statement

We recently developed a differentiation method based on rosette neural aggregates (termed RONAs) to generate balanced excitatory and inhibitory neuronal networks from human induced pluripotent stem cells (hiPSCs). It is not yet known whether human neurons derived from this method can survive and function following transplantation into an intact rat brain. To address this question, we studied the properties of grafted hiPSC-derived neurons labeled with RFP, which display stereotypical neuronal behavior, including firing and excitatory and inhibitory synaptic activity, and receive synaptic inputs from host neurons.

## Introduction

Human induced pluripotent stem cells (hiPSCs; Takahashi et al., 2007a,b; Yu et al., 2007) can be differentiated and directed to specific neuronal subtypes (Ben-Hur et al., 2004; Perrier et al., 2004; Li et al., 2005; Yan et al., 2005; Johnson et al., 2007; Lee et al., 2007; Hargus et al., 2010; Hu et al., 2010), which offers novel opportunities for modeling human neurologic diseases (Perrier et al., 2004; Roy et al., 2006; Yang et al., 2008; Hargus et al., 2010) and potentially replacement therapies. After transplantation, the spontaneous fate determination of human neuronal progenitors and their functional integration into existing circuitry is the prerequisite for their long-term therapeutic potential.

The human cerebral cortex is characterized by the diversity of cortical neurons as well as the dynamic interconnection between excitatory pyramidal neurons and inhibitory interneurons (Rakic, 2009; Lodato et al., 2011; Lui et al., 2011). In terms of modeling the development of human cortex, prior *in vitro* studies have made considerable advances in generating excitatory (glutamatergic) projection neurons or inhibitory (GABAergic) interneurons (Paşca et al., 2011; Shi et al., 2012; Liu et al., 2013; Maroof et al., 2013; Nicholas et al., 2013; Espuny-Camacho et al., 2017). Subsequently, recent studies addressed that hiPSC-derived neurons possess the ability to fire high-frequency action potentials (APs), and are capable of exhibiting spontaneous postsynaptic currents after *in vivo* grafting (Weick et al., 2011; Nicholas et al., 2013; Espuny-Camacho et al., 2017). However, there has been little focus on producing a balanced network of both excitatory and inhibitory neurons resembling the complex constitution of human cortex. We recently established a human cortical neuron culture system that has representation of all six cortical layers with both excitatory and inhibitory neuronal networks (Xu et al., 2016). It is not known whether these hiPSC-derived excitatory and inhibitory networks can survive and develop *in vivo*, and how they exert their physiologic roles in host brain.

For this study, we generated a hiPSC cell line that constitutively expresses RFP to investigate the integration properties of human neurons after transplantation into the neonatal rat brain. We show that hiPSC-derived neurons are successfully introduced into the rodent cortex. More importantly, the grafted neurons are capable of firing, receiving synaptic inputs from neighbor neurons, as well as displaying both excitatory and inhibitory synaptic responses.

## Materials and Methods

### hiPSC culture and neural differentiation

hiPSC lines were maintained on inactivated mouse embryonic fibroblasts (MEFs). To reliably visualize and trace transplanted cells *in vivo*, a stable hiPSC dsRED-SC1014 cell line was established by nucleofection with *piggybac*-dsRED transposon and *piggyback* transposase. All cell lines were maintained according to a standard protocol. Briefly, hiPSCs were cultured in human ES cell medium containing DMEM/F12 (Invitrogen), 20% knock-out serum replacement (Invitrogen), 4 ng/ml FGF2 (PeproTech), 1 mm Glutamax (Invitrogen), 100 μm nonessential amino acids (Invitrogen), 100 μm 2-mercaptoethanol (Invitrogen). Medium was changed daily. Cells were passaged using collagenase (1 mg/ml in DMEM/F12) at a ratio of 1:6–1:12. Neural differentiation of hiPSCs was based on the rosette neural aggregates (RONAs) method (Xu et al., 2016). Briefly, to initiate differentiation, hiPSC colonies were allowed to incubate with collagenase (1 mg/ml in DMEM/F12) in the incubator for ∼5-10 min. After the colony borders began to peel away from the plate, the collagenase was gently washed off the plate with growth medium. While the colony center remained attached, the colonies were selectively detached with the MEFs undisturbed. Detached hiPSC colonies were then grown as suspensions in human ES cell medium without FGF2 for 2 d in low-attachment six-well plates (Corning). From day 2 to day 6, Noggin (50 ng/ml; R&D system) or dorsomorphin (1 μm; Tocris) and SB431542 (10 μm; Tocris) were supplied in human ES cell medium (without FGF2, defined as KoSR medium). On day 7, free-floating embryoid bodies (EBs) were transferred to Matrigel- or Laminin-precoated culture plates to allow the complete attachment of EB aggregates with the supplement of N2-induction medium (NIM) containing DMEM/F12 (Invitrogen), 1% N2 supplement (Invitrogen), 100 μm MEM nonessential amino acids solution (Invitrogen), 1 mm Glutamax (Invitrogen), and heparin (2 μg/ml, Sigma-Aldrich). Cultures were continuously fed with N2 medium every other day from day 7 to 12. From day 12, N2 induction medium was changed every day. Attached aggregates broke down to form a monolayer colony on days 8–9 with typical neural-specific rosette formation. With the extension of neural induction, highly compact three-dimensional column-like neural aggregates RONAs formed in the center of attached colonies. RONAs were manually microisolated, taking special care to minimize the contaminating peripheral monolayer of flat cells and cells underneath RONAs. RONA clusters were collected and maintained as neurospheres in Neurobasal medium (Invitrogen) containing B27 minus VitA (Invitrogen) and1 mm Glutamax (Invitrogen) for 1 d. The next day, neurospheres were dissociated into single cells and plated on laminin/poly-d-lysine-coated plates for additional experiments. For neuronal differentiation, retinoic acid (RA) (2 μm), SHH (50 ng/ml), or purmorphamine (2 μm), or the combination of RA, SHH, and purmorphamine were supplemented in neural differentiation medium containing Neurobasal/B27 (Invitrogen), brain-derived neurotrophic factor (BDNF; 20 ng/ml; PeproTech), glial cell line-derived neurotrophic factor (GDNF; 20 ng/ml; PeproTech), ascorbic acid (0.2 mm; Sigma-Aldrich), and dibutyryl cAMP (0.5 mm; Sigma-Aldrich) at indicated times after neurospheres were dissociated into single cells. For long-term neuronal culture, neural differentiation medium containing rat astrocyte-conditioned Neurobasal medium/B27, BDNF, GDNF, ascorbic acid, and dibutyryl cAMP was used for maintenance.

### Animals and transplantation

Animals were housed and treated in accordance with the National Institutes of Health (NIH) *Guide for the Care and Use of Laboratory Animals* and Institutional Animal Care and Use Committees. A total of 12 neonatal rats (6 males and 6 females at postnatal day 1) were used as transplant recipients. To avoid immunosuppression, NIH nude rats (Charles River Laboratories; RRID:RGD_2312499; Liang et al., 1997) were selected. hiPSC-derived neural progenitors were manually dissociated at day 31–32 in culture. Each newborn rat received an injection of 200,000 cells. Cells were injected into the right cortex (2.0 mm posterior and 1.9 mm lateral to bregma, 2.6 mm below the dura). Analyses were performed at 10 weeks after transplantation.

### Immunostaining

For immunocytochemistry analysis, cultured cells were washed in PBS and fixed in 4% paraformaldehyde for 15 min. For immunohistochemistry, rat brain tissues were sectioned (25 μm) using a cryostat (CM3050, Leica) and collected on SuperFrost Plus glass slides (Roth). After blocking with 10% (v/v) donkey serum and 0.2% (v/v) Triton X-100 in PBS, cells and sections were incubated overnight at 4°C with primary antibodies, followed by incubations with secondary antibody (Invitrogen) for 1 h. After staining, coverslips were mounted on glass slides, and sections were coverslipped using ProLong Gold Antifade Reagent (Invitrogen). The primary antibodies used in this study were human-specific NES (Nestin; MAB5326, Millipore; RRID:AB_11211837), TBR1 (T-box brain protein 1; 1; ab31940, Abcam; RRID:AB_2200219), CTIP2 (chicken ovalbumin upstream promoter-transcription factor interacting protein 2; ab18465, Abcam; RRID:AB_2064130), BRN2 (brain-2; sc-6029, Santa Cruz Biotechnology; RRID:AB_2167385), SATB2 (special AT-rich sequence-binding protein 2; ab51502, Abcam; RRID:AB_882455), PROX1 (prospero homeobox protein 1; ab37128, Abcam; RRID:AB_882189), TUJ1 (MAB1637, Millipore; RRID:AB_2210524), and MAP2 (Microtubule-associated protein 2; M2320, Sigma-Aldrich; RRID:AB_609904; AB5622, Millipore; RRID:AB_91939). The following cyanine (Cy) 2-, Cy3-, or Cy5-conjugated secondary antibodies were used to detect the following primary antibodies: donkey antibody against mouse, donkey antibody against rat, donkey antibody against goat, and donkey antibody against rabbit (Invitrogen).

### Patch-clamp recordings in cell culture

Whole-cell recordings in hiPSC-derived cell cultures were performed at 10 weeks after neuronal differentiation. Cultures were perfused at 2 ml/min at 32°C with artificial CSF (ACSF) solution. Patch pipettes (3–5 MΩ) were filled with a pipette solution containing the following (in mm): K-gluconate 126, KCl 8, HEPES 20, EGTA 0.2, NaCl 2, MgATP 3, and Na3GTP 0.5, pH 7.3, 290–300 mOsm. Pipette resistance was 5–7 MΩ, and series resistance was typically 10–30 MΩ. The holding potential for voltage-clamp experiments was −70 mV. Action potentials were induced by a series of hyperpolarizing and depolarizing step currents. Sodium and potassium currents were evoked by a series of voltage steps (from −100 to +60 mV in 20 mV steps). Spontaneous miniature EPSCs (mEPSCs) were obtained with voltage-clamp configuration, in the presence of tetrodoxin (TTX; 1 μm; Tocris), picrotoxin (10 μm; Tocris), and bicuculline (Bic; 10 μm; Tocris). Similar procedures were used to record spontaneous miniature IPSCs (mIPSCs). The intracellular solution contained the following (in mm): Cs gluconate 122.5, CsCl 17.5, HEPES 10, EGTA 0.2, NaCl 8, MgATP 2, and Na_3_GTP 0.3, pH 7.2, 290–300 mOsm. mIPSCs were recorded in the presence of TTX (1 μm), 6-cyano-7-nitroquinoxaline-2,3-dione (CNQX; 20 μm; Tocris), and d(−)-2-amino-5-phosphonopentanoic acid (d-AP5; 50 μm; Tocris) to elicit action potentials and excitatory postsynaptic currents. Data were collected using PatchMaster software (HEKA Elektronik), sampled at 10 kHz and filtered at 2.9 kHz, then analyzed with Clampfit and Synaptosoft software.

### Electrophysiological recording of brain slices

Rats (*n* = 6) were anesthetized with isoflurane and decapitated. Transverse brain slices of 350 μm thickness were prepared at 10 weeks after differentiated hiPSC cells injection using a vibratome (VT1200S, Leica). Slices were incubated in ACSF containing the following (in mm): NaCl 125, KCl 2.5, MgSO4 1, NaH_2_PO_4_ 1.25, NaHCO3 26, CaCl2 2, and d-glucose 10. Slices were maintained in ACSF and continuously bubbled with 95% O_2_ and 5% CO_2_, first at 34°C for 30 min, and then at room temperature. A single slice was transferred into a submerged recording chamber and perfused constantly with carbogen-equilibrated ACSF at a rate of 2 ml/min. Injected human neurons expressing RFP were visualized under a 40× water immersion objective by fluorescence and differential interference contrast optics (Carl Zeiss). Recordings were performed at 32°C. For whole-cell patch-clamp studies, borosilicate glass pipettes (BF-150, Sutter Instruments) with a tip resistance of 3–5 MΩ were pulled on a Flaming-Brown micropipette puller (P-1000, Sutter Instruments) and filled with solution containing the following (in mm): K-gluconate 126, KCl 8, HEPES 20, EGTA 0.2, NaCl 2, MgATP 3, and Na3GTP 0.5, pH 7.3, 290–300 mOsm. RFP^+^ human neurons were randomly selected for recording 300–1000 μm from the graft site. Resting membrane potential (RMP) was recorded in current-clamp mode at 0 pA immediately after establishing whole-cell configuration. Series resistance (Rseries) and input resistance (Rin) were calculated from a 5 mV pulse and monitored throughout the experiment. Unstable recordings (>10% fluctuation of Rseries value) during the course of the experiments were rejected from further analysis. For voltage -clamp experiments, the membrane potential was typically held at −70 mV. Drugs were applied through a gravity-driven drug delivery system (VC-6, Warner). All recordings were obtained using a HEKA EPC10 amplifier (HEKA Elektronik), sampled at 10 kHz, and filtered at 2.9 kHz. Data were acquired by PatchMaster software (HEKA Elektronik). Na^+^ and K^+^ currents and action potentials were analyzed using Clampfit 10.5 software (Molecular Devices). Spontaneous synaptic events were analyzed using MiniAnalysis software (Synaptosoft).

### Statistical analysis

The number of cells and the number of mice used for each experiment are listed in the figure legends. Quantification is presented as the mean value with SEM, unless otherwise stated. Statistical analysis was performed using GraphPad Prism 8. Comparison between conditions was determined by a Student’s *t* test. Results were considered significant at *p* < 0.05.

## Results

### Conversion of RFP-expressing hiPSCs to neural lineage

**Figure 1.**
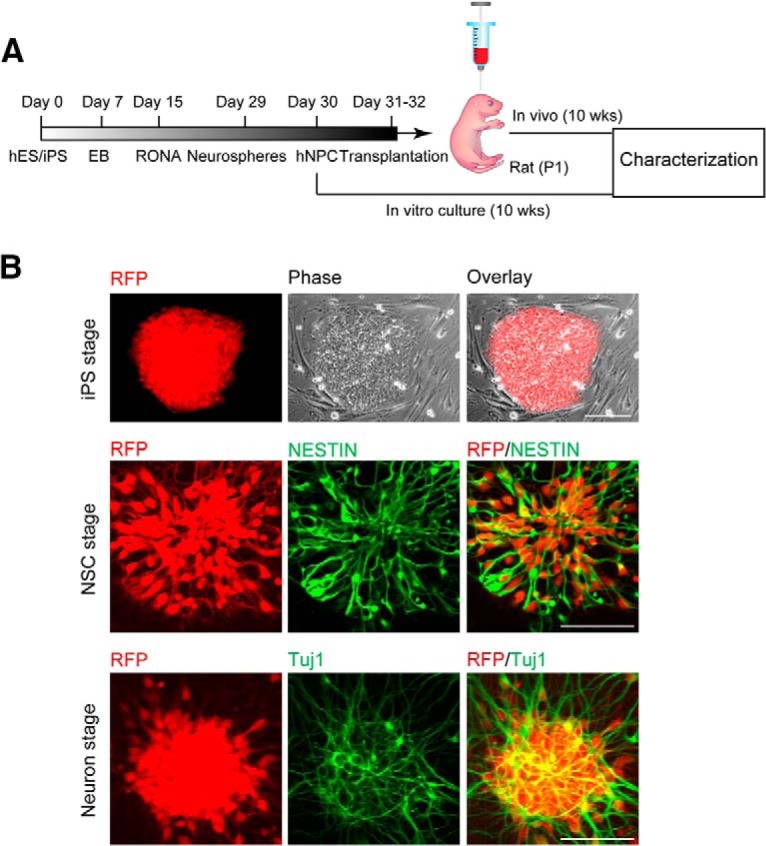
hiPSC-derived neuronal progenitors constitutively expressing RFP. ***A***, An outline showing generation and transplantation of forebrain progenitors from hiPSCs. ***B***, Establishment of stable hiPSC dsRED-SC1014 cell line in culture. Representative images showed that cells went through iPS stage, neuron stem cell (NSC) stage, and neuron stage. Cells expressed Nestin and β-tubulin III (TuJ1) after neuronal differentiation.

To facilitate the identification of hiPSC-derived neurons after transplantation, the stable hiPSC dsRED-SC1014 cell line was established by nucleofection with *piggybac*-dsRED transposon and *piggyback* transposase. Adapting our previous culture conditions (Xu et al., 2016), the cells were prepared and grown as EB suspensions until day 7. We induced differentiation of hiPSCs into forkhead box G1 forebrain progenitors to further generate excitatory and inhibitory neurons. Forebrain neural precursor cells (NPCs) were produced from RONAs at day 30, then grafted into the cortex of rat neonates (200,000 cells/injection) and analyzed after 10 weeks post-transplantation (Fig. 1*A*). Meanwhile, *in vitro* cultures were maintained until the same time point for characterization (Fig. 1*A*). The onset of neural differentiation was assessed by the expression of neuronal progenitor cell markers Nestin and β-tubulin III (TuJ1; Fig. 1*B*).

### Identity of forebrain neurons derived from hiPSCs in culture

**Figure 2.**
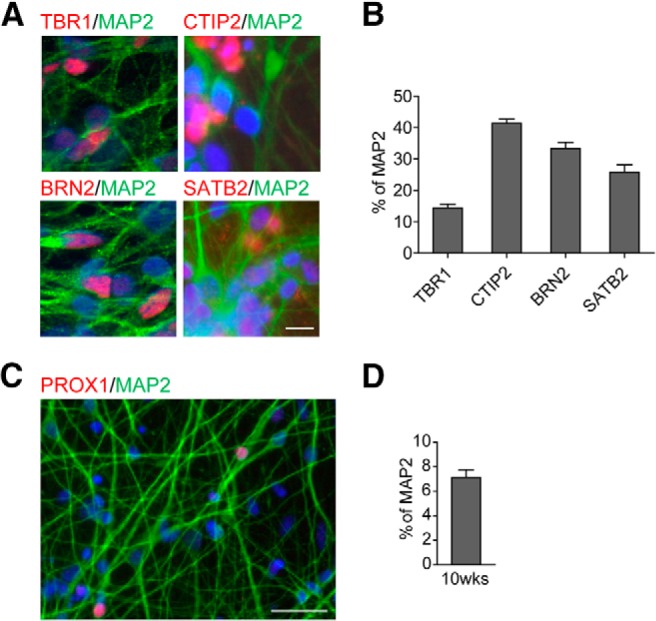
Human cortical identity of hiPSC-derived neurons in culture. ***A***, Ten weeks after differentiation, cells expressed the cortical layer-specific markers TBR1, CTIP2, BRN2, and SATB2. DAPI expression is shown in blue. Scale bar, 20 μm. ***B***, Quantification of the percentages of TBR1, CTIP2, BRN2, and SATB2 in neuronal culture. Data are expressed as the mean ± SEM from three independent experiments. ***C***, Ten weeks after differentiation, some cells expressed the hippocampal marker PROX1. DAPI expression is shown in blue. Scale bar, 100 μm. ***D***, Quantification of the percentage of PROX1 in neuronal culture. Data are expressed as the mean ± SEM (*n* = 3).

To determine the identity of hiPSC-derived neurons in culture conditions, 10 weeks after differentiation an immunocytochemical study was performed for a set of cortical-specific markers. Robust expression of TBR1 (a marker for cortical layer I, V, and VI) and CTIP2 (a marker for cortical layer V and VI), which colocalized with MAP2 were detected (Fig. 2*A*,*B*). The cultures also expressed markers for cortical layer II to V BRN2 and SATB2 (Fig. 2*A*,*B*). In addition, PROX1 was also found in cells (Fig. 2*C*,*D*). PROX1 is known to be expressed in hippocampal neurons as well as muscle satellite cells, and the MAP2/PROX1 costaining indicated the differentiation of hiPSC cells into hippocampal neurons. These data indicated that hiPSCs efficiently converted to a population of neural progenitors that corresponded to a forebrain neuronal identity.

### Balanced excitatory and inhibitory synaptic activity of hiPSC-derived neurons in culture

**Figure 3.**
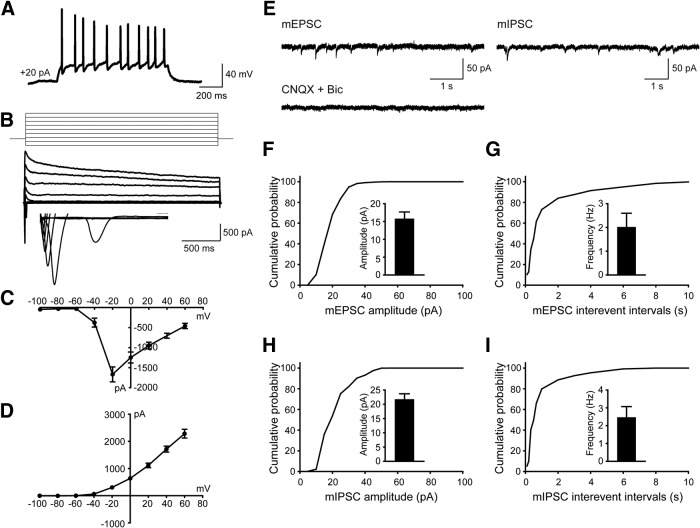
hiPSC-derived neurons display both excitatory and inhibitory synaptic activity in culture 10 weeks after differentiation. ***A***, Current-clamp recordings from hiPSCs-derived neurons showing action potential firing at depolarized potentials. ***B–D***, Representative traces showing Na^+^ (inward) and K^+^ (outward) currents (***B***) and their *I–V* curves (***C*** and ***D***) recorded from hiPSC-derived neurons (*n* = 6). ***E***, Representative traces showing mEPSCs and mIPSCs recorded in hiPSC-derived neurons, inhibited by the application of CNQX (10 µM) and Bic (20 µM). ***F***, ***G***, Amplitude (***F***) and frequency (***G***) of mEPSCs as well as their cumulative probability curves. For quantification, 18 neurons were examined, and values are expressed as the mean ± SEM. ***H***, ***I***, mIPSCs amplitude (***H***) and frequency (***I***) as well as their cumulative probability curves. For quantification, 18 neurons were examined, and values are expressed as the mean ± SEM.

To test whether the hiPSC-derived cells were functional neurons and how they contributed to neurotransmission, whole-cell patch-clamp recordings were performed to examine their electrophysiological properties 10 weeks after differentiation. Human neurons showed repetitive AP firing patterns on superthreshold current injection (Fig. 3*A*). At the single-cell level, we observed voltage-dependent sodium (Na^+^) and potassium (K^+^) currents (Fig. 3*B–D*). Notably, both mEPSCs and mIPSCs were detected in neurons (Fig. 3*E–I*), which is consistent with the notion that the balanced activity of neuronal networks was maintained by both glutamatergic synaptic outputs and GABAergic synaptic inputs (Xu et al., 2016). Overall, these data suggested that *in vitro* cortical neurogenesis from hiPSCs expressing RFP mimicked the forebrain patterning in both rodent and human.

### hiPSC-derived cortical cell grafts in rat newborn forebrain

**Figure 4.**
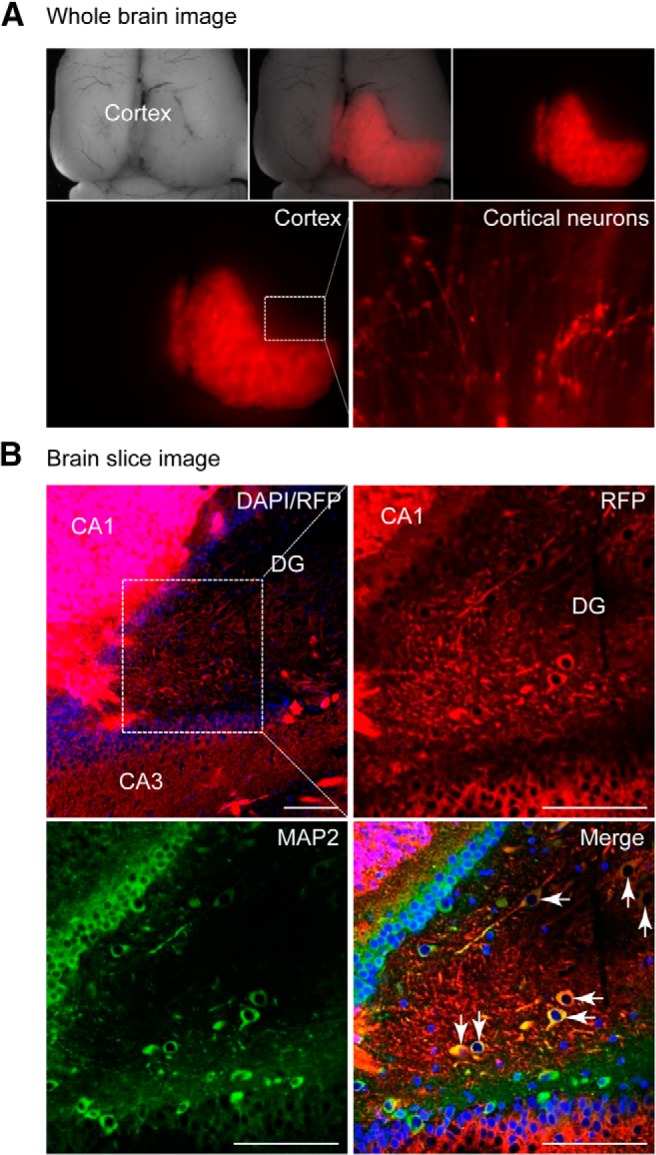
hiPSC-derived neurons integrate into the rat brain. ***A***, Rat brain image 10 weeks after introducing RFP^+^-hiPSC-NPCs (*n* = 6). RFP^+^ cortical neurons were presented. ***B***, Brain slices stained with DAPI (blue) and the mature neuronal marker MAP2 (green). Scale bar, 100 μm. CA, Cornu ammonis; DG, dentate gyrus. See also Movie 1.

Ten weeks after transplantation, immunohistochemistry revealed that the hiPSC-derived neurons were mostly retained in the forebrain (>90%) and formed axon-like neuron structures (Fig. 4*A*). Sectional analysis revealed that RFP-expressing neurons were located within the frontal cortex, including the cornu ammonis as well as the dentate gyrus, a major area undergoing neurogenesis in the adult brain, as shown by RFP/MAP2 (a mature neuronal marker) double-positive cells (Fig. 4*B*; Movie 1). Consistent with the immunostaining analysis, quantitative RT-PCR revealed the appearance of neuronal markers MAP2, β-tubulin III, and Nestin (data not shown).

Movie 1. RFP/MAP2 double-positive cells showing hiPSC-derived neurons integrated into host brain. 10.1523/ENEURO.0148-19.2019.movie.1

### Functional integration of hiPSC-derived neurons in the rat brain

**Figure 5.**
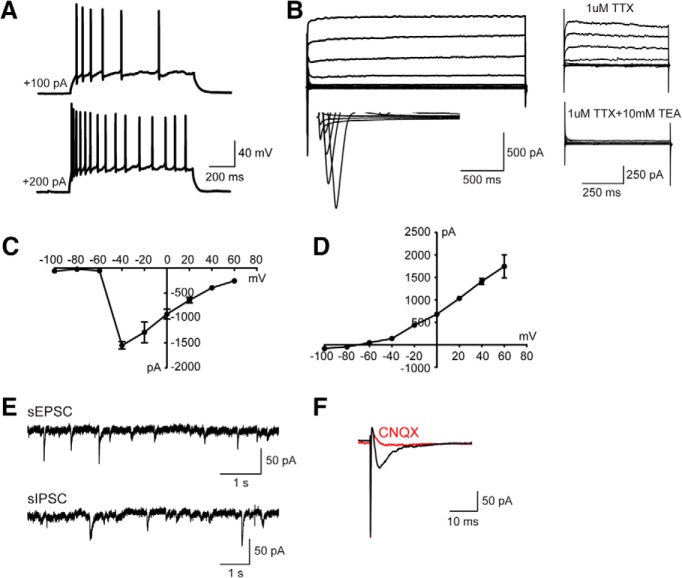
hiPSC-derived neurons functionally integrate into the synaptic circuitry of the rat brain 10 weeks after transplantation. ***A***, AP firing patterns of hiPSC-derived neurons (*n* = 14). Increased AP firing was observed with increasing current injection. ***B***, Representative traces of whole-cell Na^+^ (inward) and K^+^ (outward; *n* = 10) currents recorded from grafted cells, elicited by voltage steps from −100 mV to +60 mV in 20 mV increments, and blocked by TTX and TEA, respectively. ***C***, ***D***, *I–V* curves for voltage-gated Na^+^ (***C***) and K^+^ (***D***) currents. ***E***, Representative traces of sEPSCs (*n* = 14) and sIPSCs (*n* = 5) recorded in voltage-clamp configuration at −70 mV. sEPSCs were obtained in the presence of picrotoxin (100 μm), and sIPSCs were recorded in the presence of CNQX (20 μm) and d-AP5 (50 μm). ***F***, AMPA receptor-mediated postsynaptic currents were evoked by stimulation delivered from an electrode placed ∼200–300 μm away from the transplanted cell, which was blocked by the subsequent application of CNQX.

Furthermore, we analyzed the functionality of hiPSC-derived cells integrated into the rat cortex, using patch-clamp recordings of RFP^+^ human cells on *ex vivo* brain slices acutely obtained from rats 10 weeks following transplantation. Strikingly, we found that human neurons in the slices examined displayed passive membrane properties and excitability. Cells were capable of firing repetitive action potentials in response to depolarizing current injection, and an increase in the action potential firing rate was observed with increasing current (Fig. 5*A*). In voltage-clamp configuration, depolarizing voltage steps induced characteristic Na^+^ and K^+^ currents, which were sensitive to the voltage-gated Na^+^ channel blocker TTX, and the voltage-gated K^+^ channel blocker tetraethylammonium (TEA), respectively (Fig. 5*B–D*).

We next investigated the synaptic connectivity of the transplanted neurons. We observed spontaneous EPSCs (sEPSCs) in recorded human neurons (Fig. 5*E*), indicating that the cells had functional excitatory synapses. Importantly, spontaneous IPSCs (sIPSCs) were also detected in some transplanted neurons, suggesting that the inhibitory synapses of the cells were physiologically functional (Fig 5*E*). Moreover and most strikingly, in hiPSC-derived neurons, excitatory, AMPA receptor-mediated currents could be evoked by stimulating the intact adjacent region to the transplanted cells (Fig. 5*F*). Together, these data showed that hiPSC neurons were able to display stereotypical neuronal behavior, including firing properties, and excitatory and inhibitory synaptic activity, suggesting their ability to integrate into the host brain local neuronal circuits.

### Functional comparison of hiPSC-derived neurons in culture or from transplanted brain slices

**Figure 6.**
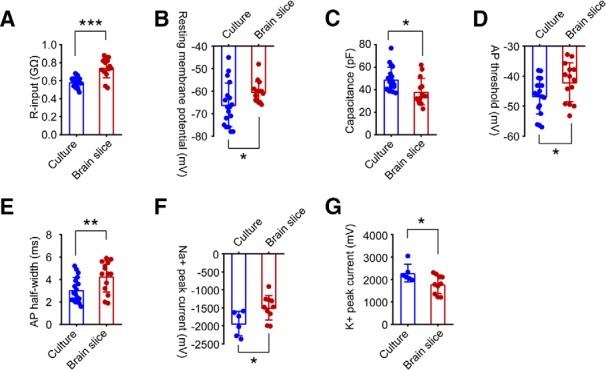
Quantification of electrical properties of hiPSC-derived neurons in culture versus in brain slices 10 weeks postdifferentiation. ***A–E***, R-input, RMP, cell capacitance, AP threshold, and AP width of hiPSC-derived neurons were measured in whole-cell patch-clamp recordings. hiPSC-derived neurons in rat brain slices (*n* = 14) showed significantly increased R-input (***A***) and RMP (***B***), decreased cell capacitance (***C***), enhanced AP threshold (***D***), and AP half-width (***E***) compared with hiPSC-derived neurons in culture (*n* = 18). Data are represented as the mean ± SEM, *p < 0.05, **p < 0.01, ***p < 0.001, Student’s *t* test. ***F***, ***G***, Quantification of Na^+^ (***F***) and K^+^ (***G***) peak currents. hiPSC-derived neurons in culture (*n* = 6) displayed enhanced Na^+^ and K^+^ peak currents compared with hiPSC-derived neurons in slices (*n* = 10). Data are expressed as the mean ± SEM, *p < 0.05, Student’s *t* test.

To assess whether there are differences in the functional activity of hiPSC-derived neurons between culture and rat brain slices, we quantified neuronal properties at the single-cell level, including input resistance (R-input), RMP, cell capacitance, AP threshold, AP half-width, as well as voltage-gated Na^+^ and K^+^ currents. At 10 weeks postdifferentiation, the average R-input values for human neurons in culture and in brain slices were 0.59 ± 0.06 and 0.74 ± 0.11 GΩ (***p = 0.0001; Fig. 6*A*), respectively. The average RMPs of hiPSC-derived neurons in culture were −66.06 ± 2.29 mV, significantly decreased compared with those of hiPSC-derived neurons in brain slices (−60.33 ± 1.21 mV; *p = 0.046; Fig. 6*B*). Human neurons in culture showed a larger average cell capacitance (49.11 ± 2.56 pF) compared with human neurons in slices (38.19 ± 3.15 pF; *p = 0.011; Fig. 6*C*). In response to steps of current injection, compared with hiPSC-derived neurons in culture, hiPSC-derived neurons in brain slices appeared to have increased AP thresholds (hiPSC-derived neurons in brain slices: −42.01 ± 1.74 mV; hiPSC-derived neurons in culture: −46.67 ± 1.41 mV; *p* = 0.044; Fig. 6*D*) and AP half-widths (hiPSC-derived neurons in brain slices: 4.28 ± 0.37 ms; hiPSC-derived neurons in culture: 3.07 ± 0.26 ms; **p = 0.001; Fig. 6*E*). Furthermore, hiPSC-derived neurons in slices showed smaller Na^+^ peak current densities (−1495 ± 107 pA) and K^+^ peak current densities (1787 ± 130 pA), than hiPSC-derived neurons in culture (−1936 ± 137 pA and 2283 ± 161 pA; *p = 0.024 and *p = 0.033; Fig. 6*F*,*G*). Together, for age-matched hiPSC-derived neurons at 10 weeks, cells in culture showed lower R-input, more negative RMP, larger cell capacitance, decreased AP threshold and AP width, and larger Na^+^ and K^+^ channel currents, compared with cells in brain slices. Our data indicated that the development and maturation of transplanted neurons derived from hiPSC cells is delayed compared with neurons in culture.

## Discussion

Here, we model functional human cortical neuron development from hiPSCs and demonstrate their acquisition of excitatory and inhibitory homeostatic features both *in vitro* and in transplanted rat brain *in vivo*. The transplanted cells retain the composition of the human cortex and integrated functionally in the host rat brain.

High brain function, as well as neurologic and neuropsychiatric diseases such as Alzheimer’s disease and schizophrenia, require a balanced neurotransmission contributed by both excitatory neurons and inhibitory interneurons (Turrigiano and Nelson, 2004; Yizhar et al., 2011). Pluripotent stem cells possess attractive features because of their capacity for large-scale expansion and their potential for differentiation into a range of neural cell types. Both *in vitro* and *in vivo* procedures for human corticogenesis from IPSCs have been extensively studied (Gaspard et al., 2008; Michelsen et al., 2015; Espuny-Camacho et al., 2017). However, establishing homeostatic neuronal networks reflective of human cortex patterning has been extremely challenging until the recently reported RONA method (Xu et al., 2016), which gives rise to a balanced human cortical neuron system composed of both glutamatergic projection neurons and a diversity of GABAergic interneurons. Consistently, neuronal markers specific for cortical layers (TBR1, CTIP2, BRN2, SATB2, and PROX1) were detected in hiPSC-derived neurons, indicating that these human neurons generated display an identity corresponding to the six layers of the human cortex. Notably, robust EPSCs and IPSCs, shown as mEPSCs and mIPSCs, were observed in human neurons labeled with RFP 10 weeks after the induction of neuronal differentiation. These data support the idea that the RONA culture method favors the formation of functional excitatory and inhibitory networks (Xu et al., 2016). Also, the ubiquitous expression of RFP does not affect the effective development of neural cells and their acquisition of electrophysiological properties.

The generation of better *in vivo* models that closely resemble the physiologic features of the human brain is critical for testing new hypotheses (Espuny-Camacho et al., 2017) and developing advanced disease models. The integration of external hiPSC-derived neuronal precursors into host circuitry is a complicated affair over a prolonged period of time, including several aspects such as temporal patterning, morphologic development, and acquisition of proper functions. In this study, our main purpose was to determine whether transplanted hiPSC-derived neuronal precursors from our differentiation protocol would lead to functional and integrated neurons. These hiPSC-derived neural precursors are able to survive and develop, and to express neuronal markers at both the morphologic and molecular level, as assessed 10 weeks post-transplantation. Importantly, grafted neurons exhibited firing properties, with increased frequency of action potential firing in response to increasing current injection. These data are consistent with previous work showing human neurons with mature electrophysiological profiles following transplantation into the telencephalon of neonatal mice (Koch et al., 2009; Denham et al., 2012) or into stroke-injured rat brain (Tornero et al., 2017). It is interesting that there are functional differences between cultured hiPSC-derived neurons versus transplanted neurons. Our results highlight that transplanted human neurons display functional neuron behaviors, but their development is developmentally delayed compared with age-matched cultured human neurons, as measured by intrinsic properties, AP threshold, and AP half-width, as well as Na^+^ and K^+^ channel currents. The reconstruction of neural circuitry and network communication between transplanted neurons with host cells might be one of the reasons. Similar observations have been reported previously (Thompson and Björklund, 2015), showing that grafted neurons generated from hiPSC cells continue to mature after 10 weeks. It is noteworthy that in our system, both EPSCs and IPSCs were detected in grafted cells, which suggests that our hiPSCs not only can be differentiated into excitatory projection neurons, but also are able to develop into interneuron subtypes and produce GABAergic outputs. Additionally, our hiPSC-derived neurons are capable of receiving synaptic inputs from host endogenous neurons. Thus, taken together the data indicate these transplanted neurons generated from hiPSC cells integrate into the host brain at a functional level. Future studies will characterize the molecular identity, the regional patterns, as well as the physiologic fate of the transplanted neurons over time.

In conclusion, we show that hiPSC-derived neurons are capable of completing synaptic integration with a pre-existing network *in vivo* and can make both excitatory and inhibitory connections with host neurons. It will be important to use our approach for modeling pathologic features present in human disease brain to explore disease etiology and to determine whether this approach can replace neurons lost to disease, stroke, or trauma as a potential new therapeutic strategy.

## Synthesis

Reviewing Editor: Dritan Agalliu, Columbia University Medical Center Decisions are customarily a result of the Reviewing Editor and the peer reviewers coming together and discussing their recommendations until a consensus is reached. When revisions are invited, a fact-based synthesis statement explaining their decision and outlining what is needed to prepare a revision will be listed below. The following reviewer(s) agreed to reveal their identity: Edmund Au.

The authors have previously published a study in Science Translational Medicine in which they describe a novel method for simultaneously generating excitatory and inhibitory neurons from FoxG1+ progenitors. They suggest that this can be achieved by timely addition of retinoic acid between days 24-30. Here, the authors claim that their mixed exc/inh cultures can xenograft in rat brain.

The study does not represent an advance since the data does not address the central question implied in the visual abstract: Is there a functional difference when their exc/inh cultures mature in host cortical circuitry? This is a major concern that the authors need to address in the revision and discuss in detail.

In addition, the study has some technical limitations as outlined below:

1) There are no statistical tests performed in the current study. The authors should directly compare 10 weeks post differentiation in culture vs. transplanted using proper statistical methods.

2) It appears that the I-V curves for Na+ and K+ are very different between 10 weeks in culture and 10 weeks transplanted. The I-V curves for 10 week cultures are also strikingly different from the 8-week cultures described in Xu et al., 2016 (Fig 4A and B). The authors should comment on these discrepancies.

3) The authors mention of -60.33 mV RMP (with a very narrow error range). That is remarkably depolarized for a 2 1/2 month old xenotransplant. Please include the number of cells recorded and their raw data points for RMP. A direct comparison with RMP for 10 week old culture would also be helpful here. Similarly, in their recordings of whole cell intrinsic physiology, the authors likely also have data for input resistance and capacitance, which are also measures of functional maturity, as are AP width and AP threshold. It would be very informative to compare these measures in cells recorded in culture vs. transplants.

3) In the methods, the authors state that 200 000 cells were injected. In Figure 4A looks it looks like the authors injected a lot more than 200 000 cells. It actually looks more like a tumor! If so, which cells were recorded in the xenografts? How far away were they from the graft site? IHC of graft site - what kinds of cells are present in huge growth present in Figure 4A?

4) In Figure 2D, Prox1 quantification is weirdly separate from the rest of the markers. Why is that? Also, given the quantification, it appears that almost all of the cells are excitatory in nature. This stands in contrast to 2016 paper results.

5) Where in the cortex were the cells transplanted? Was the rat immunosuppressed? If so how?

## References

[B1] Ben-Hur T, Idelson M, Khaner H, Pera M, Reinhartz E, Itzik A, Reubinoff BE (2004) Transplantation of human embryonic stem cell-derived neural progenitors improves behavioral deficit in Parkinsonian rats. Stem Cells 22:1246–1255. 10.1634/stemcells.2004-0094 15579643

[B2] Denham M, Parish CL, Leaw B, Wright J, Reid CA, Petrou S, Dottori M, Thompson LH (2012) Neurons derived from human embryonic stem cells extend long-distance axonal projections through growth along host white matter tracts after intra-cerebral transplantation. Front Cell Neurosci 6:11. 10.3389/fncel.2012.00011 22470319 PMC3311135

[B3] Espuny-Camacho I, Arranz AM, Fiers M, Snellinx A, Ando K, Munck S, Bonnefont J, Lambot L, Corthout N, Omodho L, Vanden Eynden E, Radaelli E, Tesseur I, Wray S, Ebneth A, Hardy J, Leroy K, Brion JP, Vanderhaeghen P, De Strooper B (2017) Hallmarks of Alzheimer's disease in stem-cell-derived human neurons transplanted into mouse brain. Neuron 93:1066–1081.e8. 10.1016/j.neuron.2017.02.001 28238547

[B4] Gaspard N, Bouschet T, Hourez R, Dimidschstein J, Naeije G, van den Ameele J, Espuny-Camacho I, Herpoel A, Passante L, Schiffmann SN, Gaillard A, Vanderhaeghen P (2008) An intrinsic mechanism of corticogenesis from embryonic stem cells. Nature 455:351–357. 10.1038/nature07287 18716623

[B5] Hargus G, Cooper O, Deleidi M, Levy A, Lee K, Marlow E, Yow A, Soldner F, Hockemeyer D, Hallett PJ, Osborn T, Jaenisch R, Isacson O (2010) Differentiated Parkinson patient-derived induced pluripotent stem cells grow in the adult rodent brain and reduce motor asymmetry in Parkinsonian rats. Proc Natl Acad Sci U S A 107:15921–15926. 10.1073/pnas.1010209107 20798034 PMC2936617

[B6] Hu BY, Weick JP, Yu J, Ma LX, Zhang XQ, Thomson JA, Zhang SC (2010) Neural differentiation of human induced pluripotent stem cells follows developmental principles but with variable potency. Proc Natl Acad Sci U S A 107:4335–4340. 10.1073/pnas.0910012107 20160098 PMC2840097

[B7] Johnson MA, Weick JP, Pearce RA, Zhang SC (2007) Functional neural development from human embryonic stem cells: accelerated synaptic activity via astrocyte coculture. J Neurosci 27:3069–3077. 10.1523/JNEUROSCI.4562-06.2007 17376968 PMC2735200

[B8] Koch P, Opitz T, Steinbeck JA, Ladewig J, Brüstle O (2009) A rosette-type, self-renewing human ES cell-derived neural stem cell with potential for in vitro instruction and synaptic integration. Proc Natl Acad Sci U S A 106:3225–3230. 10.1073/pnas.0808387106 19218428 PMC2651316

[B9] Lee H, Shamy GA, Elkabetz Y, Schofield CM, Harrsion NL, Panagiotakos G, Socci ND, Tabar V, Studer L (2007) Directed differentiation and transplantation of human embryonic stem cell-derived motoneurons. Stem Cells 25:1931–1939. 10.1634/stemcells.2007-0097 17478583

[B10] Li XJ, Du ZW, Zarnowska ED, Pankratz M, Hansen LO, Pearce RA, Zhang SC (2005) Specification of motoneurons from human embryonic stem cells. Nat Biotechnol 23:215–221. 10.1038/nbt1063 15685164

[B11] Liang SC, Lin SZ, Yu JF, Wu SF, Wang SD, Liu JC (1997) F344-rnu/rnu athymic rats: breeding performance and acceptance of subcutaneous and intracranial xenografts at different ages. Lab Anim Sci 47:549–553. 9355102

[B12] Liu Y, Weick JP, Liu H, Krencik R, Zhang X, Ma L, Zhou GM, Ayala M, Zhang SC (2013) Medial ganglionic eminence-like cells derived from human embryonic stem cells correct learning and memory deficits. Nat Biotechnol 31:440–447. 10.1038/nbt.2565 23604284 PMC3711863

[B13] Lodato S, Rouaux C, Quast KB, Jantrachotechatchawan C, Studer M, Hensch TK, Arlotta P (2011) Excitatory projection neuron subtypes control the distribution of local inhibitory interneurons in the cerebral cortex. Neuron 69:763–779. 10.1016/j.neuron.2011.01.015 21338885 PMC3061282

[B14] Lui JH, Hansen DV, Kriegstein AR (2011) Development and evolution of the human neocortex. Cell 146:18–36. 10.1016/j.cell.2011.06.030 21729779 PMC3610574

[B15] Maroof AM, Keros S, Tyson JA, Ying SW, Ganat YM, Merkle FT, Liu B, Goulburn A, Stanley EG, Elefanty AG, Widmer HR, Eggan K, Goldstein PA, Anderson SA, Studer L (2013) Directed differentiation and functional maturation of cortical interneurons from human embryonic stem cells. Cell Stem Cell 12:559–572. 10.1016/j.stem.2013.04.008 23642365 PMC3681523

[B16] Michelsen KA, Acosta-Verdugo S, Benoit-Marand M, Espuny-Camacho I, Gaspard N, Saha B, Gaillard A, Vanderhaeghen P (2015) Area-specific reestablishment of damaged circuits in the adult cerebral cortex by cortical neurons derived from mouse embryonic stem cells. Neuron 85:982–997. 10.1016/j.neuron.2015.02.001 25741724

[B17] Nicholas CR, Chen J, Tang Y, Southwell DG, Chalmers N, Vogt D, Arnold CM, Chen YJ, Stanley EG, Elefanty AG, Sasai Y, Alvarez-Buylla A, Rubenstein JL, Kriegstein AR (2013) Functional maturation of hPSC-derived forebrain interneurons requires an extended timeline and mimics human neural development. Cell Stem Cell 12:573–586. 10.1016/j.stem.2013.04.005 23642366 PMC3699205

[B18] Paşca SP, Portmann T, Voineagu I, Yazawa M, Shcheglovitov A, Paşca AM, Cord B, Palmer TD, Chikahisa S, Nishino S, Bernstein JA, Hallmayer J, Geschwind DH, Dolmetsch RE (2011) Using iPSC-derived neurons to uncover cellular phenotypes associated with Timothy syndrome. Nat Med 17:1657–1662. 10.1038/nm.2576 22120178 PMC3517299

[B19] Perrier AL, Tabar V, Barberi T, Rubio ME, Bruses J, Topf N, Harrison NL, Studer L (2004) Derivation of midbrain dopamine neurons from human embryonic stem cells. Proc Natl Acad Sci U S A 101:12543–12548. 10.1073/pnas.0404700101 15310843 PMC515094

[B20] Rakic P (2009) Evolution of the neocortex: a perspective from developmental biology. Nat Rev Neurosci 10:724–735. 10.1038/nrn2719 19763105 PMC2913577

[B21] Roy NS, Cleren C, Singh SK, Yang L, Beal MF, Goldman SA (2006) Functional engraftment of human ES cell-derived dopaminergic neurons enriched by coculture with telomerase-immortalized midbrain astrocytes. Nat Med 12:1259–1268. 10.1038/nm1495 17057709

[B22] Shi Y, Kirwan P, Smith J, Robinson HP, Livesey FJ (2012) Human cerebral cortex development from pluripotent stem cells to functional excitatory synapses. Nat Neurosci 15:477–486. 10.1038/nn.3041 22306606 PMC3882590

[B23] Takahashi K, Okita K, Nakagawa M, Yamanaka S (2007a) Induction of pluripotent stem cells from fibroblast cultures. Nat Protoc 2:3081–3089. 10.1038/nprot.2007.418 18079707

[B24] Takahashi K, Tanabe K, Ohnuki M, Narita M, Ichisaka T, Tomoda K, Yamanaka S (2007b) Induction of pluripotent stem cells from adult human fibroblasts by defined factors. Cell 131:861–872. 10.1016/j.cell.2007.11.019 18035408

[B25] Thompson LH, Björklund A (2015) Reconstruction of brain circuitry by neural transplants generated from pluripotent stem cells. Neurobiol Dis 79:28–40. 10.1016/j.nbd.2015.04.003 25913029

[B26] Tornero D, Tsupykov O, Granmo M, Rodriguez C, Grønning-Hansen M, Thelin J, Smozhanik E, Laterza C, Wattananit S, Ge R, Tatarishvili J, Grealish S, Brüstle O, Skibo G, Parmar M, Schouenborg J, Lindvall O, Kokaia Z (2017) Synaptic inputs from stroke-injured brain to grafted human stem cell-derived neurons activated by sensory stimuli. Brain 140:692–706. 10.1093/brain/aww347 28115364

[B27] Turrigiano GG, Nelson SB (2004) Homeostatic plasticity in the developing nervous system. Nat Rev Neurosci 5:97–107. 10.1038/nrn1327 14735113

[B28] Weick JP, Liu Y, Zhang SC (2011) Human embryonic stem cell-derived neurons adopt and regulate the activity of an established neural network. Proc Natl Acad Sci U S A 108:20189–20194. 10.1073/pnas.1108487108 22106298 PMC3250161

[B29] Xu JC, Fan J, Wang X, Eacker SM, Kam TI, Chen L, Yin X, Zhu J, Chi Z, Jiang H, Chen R, Dawson TM, Dawson VL (2016) Cultured networks of excitatory projection neurons and inhibitory interneurons for studying human cortical neurotoxicity. Sci Transl Med 8:333–348. 10.1126/scitranslmed.aad0623 PMC5595216 27053772

[B30] Yan Y, Yang D, Zarnowska ED, Du Z, Werbel B, Valliere C, Pearce RA, Thomson JA, Zhang SC (2005) Directed differentiation of dopaminergic neuronal subtypes from human embryonic stem cells. Stem Cells 23:781–790. 10.1634/stemcells.2004-0365 15917474 PMC2707939

[B31] Yang D, Zhang ZJ, Oldenburg M, Ayala M, Zhang SC (2008) Human embryonic stem cell-derived dopaminergic neurons reverse functional deficit in parkinsonian rats. Stem Cells 26:55–63. 10.1634/stemcells.2007-0494 17951220 PMC2707927

[B32] Yizhar O, Fenno LE, Prigge M, Schneider F, Davidson TJ, O'Shea DJ, Sohal VS, Goshen I, Finkelstein J, Paz JT, Stehfest K, Fudim R, Ramakrishnan C, Huguenard JR, Hegemann P, Deisseroth K (2011) Neocortical excitation/inhibition balance in information processing and social dysfunction. Nature 477:171–178. 10.1038/nature10360 21796121 PMC4155501

[B33] Yu J, Vodyanik MA, Smuga-Otto K, Antosiewicz-Bourget J, Frane JL, Tian S, Nie J, Jonsdottir GA, Ruotti V, Stewart R, Slukvin II, Thomson JA (2007) Induced pluripotent stem cell lines derived from human somatic cells. Science 318:1917–1920. 10.1126/science.1151526 18029452

